# Newer nerve transfers

**DOI:** 10.1186/1753-6561-9-S3-A22

**Published:** 2015-05-19

**Authors:** Hari Venkatramani

**Affiliations:** 1 Ganga Hospital, Coimbatore, 641043, India

## 

In nerve transfer, we transfer a physiologically active nerve to a distal, more important but irreparably paralyzed nerve. This surgical procedure is best done early within 6 months of the injury. Nerve transfer can be broadly classified into four categories: (1) Extraplexal; (2) Intraplexal and (3) Targeted nerve transfer.

Prime extraplexal donors are the spinal accessory, phrenic and intercostal nerves. There are many combination of nerve transfer, depending upon the loss of action and available motors.

For shoulder abduction: 1) Spinal accessory to supra-scapular: this transfer can be done from a dorsal approach; 2) Triceps branch of radial nerve to axillary from an anterior approach

For elbow flexion: 1) Spinal accessory to biceps and brachialis split sural nerve transfer: this extraplexal transfer involves using a reversed sural nerve graft and the two ends are used for the biceps and brachialis separately; 2) Ulnar to brachialis and median to biceps

For Elbow extension: 1) Ulnar nerve to triceps; 2)Intercostal to Triceps

For wrist extension: Median nerve to ECRB branch of Radial nerve

For Finger extension: Transferring the FDS branch of median to posterior interosseous nerve

For distal Ulnar nerve neurotisation to prevent Claw deformity and sensation:

Pronator quadratus branch of median to deep motor branch of Ulnar nerve

